# Patient’s thoughts and expectations about centres of expertise for PKU

**DOI:** 10.1186/s13023-020-01647-7

**Published:** 2021-01-06

**Authors:** A. M. J. van Wegberg, A. MacDonald, D. Abeln, T. S. Hagedorn, E. Lange, F. Trefz, D. van Vliet, F. J. van Spronsen

**Affiliations:** 1 Department of Metabolic Diseases, Beatrix Children’s Hospital, University Medical Centre Groningen, University of Groningen, 9713 GZ Groningen, The Netherlands; 2 Dietetic Department, Birmingham Children’s Hospital, Birmingham, B4 6NH UK; 3 Dutch Society for PKU, Tiel, The Netherlands; 4 Deutsche Interessengemeinschaft Phenylketonurie, Fürth, Germany; 5 National Society for Phenylketonuria United Kingdom, Preston, UK; 6 University Children’s Hospital, Dietmar Hopp Metabolic Centre, 69120 Heidelberg, Germany

**Keywords:** Phenylketonuria, Patients view, Centre of expertise, European reference network

## Abstract

**Background:**

In the Netherlands (NL) the government assigned 2 hospitals as centres of expertise (CE) for Phenylketonuria (PKU), while in the United Kingdom (UK) and Germany no centres are assigned specifically as PKU CE’s.

**Methods:**

To identify expectations of patients/caregivers with PKU of CEs, a web-based survey was distributed through the national Phenylketonuria societies of Germany, NL and UK.

**Results:**

In total, 105 responded (43 patients, 56 parents, 4 grandparents, 2 other) of whom 59 were from NL, 33 from UK and 13 from Germany. All participants (n = 105) agreed that patients and/or practitioners would benefit from CEs. The frequency patients would want to visit a CE, when not treated in a CE (n = 83) varied: every hospital visit (24%, n = 20), annual or bi-annual (45%, n = 37), at defined patient ages (6%, n = 5), one visit only (22%, n = 18), or never (4%, n = 3). Distance was reported as a major barrier (42%, n = 35). 78% (n = 65) expected CE physicians and dieticians to have a higher level of knowledge than in non-CE centres. For participants already treated in a CE (n = 68), 66% requested a more extensive annual or bi-annual review. In general, psychology review and neuropsychologist assessment were identified as necessary by approximately half of the 105 participants. In addition, 66% (n = 68) expected a strong collaboration with patient associations.

**Conclusion:**

In this small study, most participants expected that assigning CEs will change the structure of and delivery of Phenylketonuria care.

## Introduction

Phenylketonuria (PKU; McKusick #261600) is a rare autosomal recessive inborn error of phenylalanine (Phe) metabolism, caused by a deficiency in the hepatic enzyme phenylalanine hydroxylase (PAH) [1]. As with many rare diseases, patients across countries and within the same countries, do not have equality of access to specialized diagnostics, treatment or care [2]. To improve access to care, in 2011, the European parliament supported cross-border healthcare by giving patients the right to receive medical treatment in another EU member state (Directive 2011/24/EU). To further improve access to care, European Reference Networks (ERNs) were created in 2017, through collaboration with the European Union, physicians and patients. An ERN is a virtual network consisting of centres of expertise (CE) distributed throughout Europe. CEs must meet specific criteria and conditions to become part of an ERN (2014/286/EU). The ERN specific for inherited metabolic disorders (MetabERN) aims to connect CEs across Europe to promote prevention, accelerate diagnosis and improve standards of care for patients living with these disorders.

In the Netherlands, the Dutch minister of Health, Welfare and Sport appointed 2 of 6 metabolic centres (University Medical Centre Groningen and Amsterdam University Medical Centre) as CE’s for PKU specifically because of their multidisciplinary care, leading PKU scientific research and specific quality criteria [3, 4]. In order to be considered as a CE, Hannerman-Weber et al. [5] emphasises centres must combine operational care with explorative activities. Indeed, both Dutch CEs for PKU are affiliated with the MetabERN.

Following the ERN structure, CEs in PKU should have assessed all patients with PKU in their designated area. This means that all patients should receive care from a CE, without the CE necessarily taking over routine care from the local metabolic treatment centres. In contrast, Germany and the United Kingdom (UK), do not appoint CEs in PKU. From 2013, Germany has developed a three-tiered structure for treating rare disorders including appointing all metabolic centres as CEs. However, not one centre is identified as providing additional expertise in a specific inborn error of metabolism [6]. In the UK, there are centres who provide speciality services to patients with inherited metabolic disorders, including PKU, but like Germany no one centre is identified as expert in PKU care. The thoughts and expectations of patients and caregivers about CEs have not been explored. With this study we aimed to evaluate patients and caregiver understanding, expectations and barriers about PKU care from a CE.

## Materials and methods

### Questionnaire development

A Dutch web-based survey was developed in 2017 together with the participation from 6 patients with PKU and/or caregivers, recruited by the Dutch PKU society. After 3 feedback rounds the survey was finalized. After the Dutch data collection was completed, in 2018, the Dutch survey was translated into English and German to increase the patient numbers and compare with other countries. The questionnaire (translated to English) is added as Additional file 1. The questionnaire consisted of 19 closed, semi-closed and open questions. There was one difference in the design for the Dutch questionnaire. CEs had already been introduced in the Netherlands, so questions were specific for this circumstance. Using adaptive questioning, Dutch participants answered 15 questions. The UK and German participants had to ‘picture’ their care either in a CE or a general metabolic treatment centre and had to answer 19 questions. The web-based surveys were built using the software Qualtrics (https://www.qualtrics.com/). The technical functionality was tested before the survey was distributed.

### Distribution

The survey was open to any PKU patient or caregiver with access to the anonymous survey link. The Dutch survey-link was distributed in March 2017 by the Dutch PKU society through email, post, and social media. In the UK, the survey-link was distributed in October 2018 by the UK PKU patient group (NSPKU) through social media and newsletters. In Germany, the survey-link was distributed in December 2018 by the German PKU society (DIG-PKU) through social media, their website and was additionally promoted during their annual members meeting. To increase the number of participants several reminders were sent by all three patient organisations. Surveys were open for 4 to 6 months.

### Data analysis

**Fig. 1:**
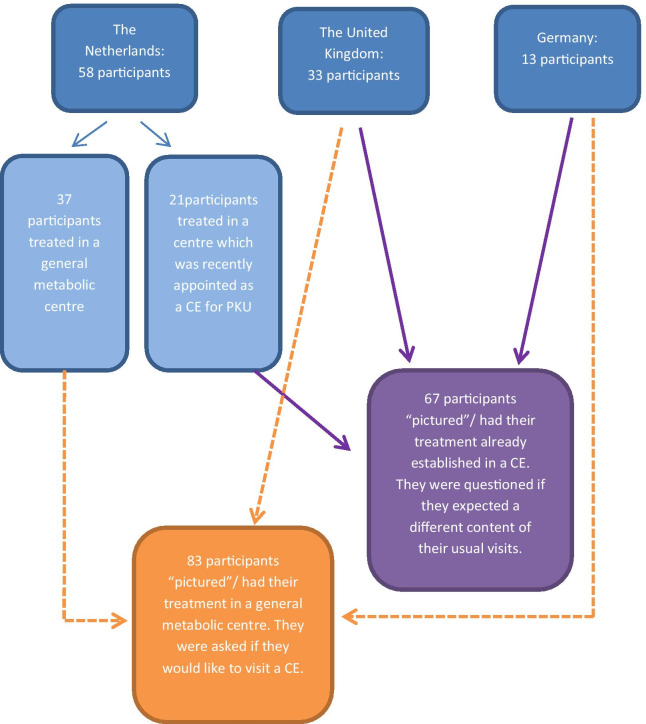
Flowchart of the participants. In the Netherlands, the Dutch minister of Health, Welfare and Sport appointed 2 of 6 PKU centres as CE’s. In contrast, Germany and the UK, do not appoint CEs in PKU. Participants from the Netherlands only answered the questions specific for their condition. Participants form the UK and Germany were asked to imagine both situations

Data were collected from March 2017 to June 2019 and were saved anonymously. Only complete questionnaires were analysed. IP addresses, location data (latitude and longitude), and the general information of the first set of questions was checked to control for double entries from the same individual, Data was analyzed in SPSS version 23. Only descriptive data is presented. Data are presented separately for patients who were treated or ‘pictured’ treatment in a CE (n = 68) versus a general metabolic treatment centre (n = 83, Fig. 1).

### Ethics

The questionnaire did not collect personal data. Ethical consent was not sought as it was clarified at the beginning of the questionnaire that the primary purpose of the survey was to gain opinion about the role and benefit of CE centres to support patients with PKU. It was also stated that the data would be saved in an anonymized form. Adults with PKU and caregivers gave their consent by their voluntary completion and submission of the online questionnaire.

## Results

### Responses

In total 202 responses were recorded (102 from the Netherlands, 81 from UK and 19 from Germany). Of these 202 responses, 47 were completely blank, 48 were incomplete and 2 responses were not PKU related. Of these 105 completed responses, one duplicate entry was removed. At the time of distribution the Dutch PKU society counted 463 individual members (1 membership per family), the NSPKU counted > 700 members (1 membership per family) and the DIG-PKU counted 1665 individual members (including multiple memberships per family). However, it is unclear how many of them had access to social media.

### Participants

In total, 104 participants completed the survey (42 patients, 56 parents, 4 grandparents and 2 others). Fifty-eight participants were from the Netherlands, 33 from UK and 13 from Germany. Eighty-five (82%) of patients followed a protein restricted diet with protein substitutes whereas 9 (9%) were prescribed tetrahydrobiopterin (BH4) without or combined with dietary treatment (Table 1).

**Table 1:** Characteristics of PKU patients provided by the correspondents

	Self-reported details of the PKU patients (n = 42)	Details of the PKU children of the parents/caregivers (n = 62)^a^
*Age*		
Median age	35 years	10 years
Age range	8–62 years^b^	0–60 years ^c,d^
*Gender*		
Male	21.4% (n = 9)	19.4% (n = 12)
Female	78.6% (n = 33)	80.6% (n = 50)
*Treatment*		
Protein restricted diet with amino acid supplements/ GMP	85.7% (n = 36)	79% (n = 49)
Protein restricted diet without amino acid supplements/GMP	0% (n = 0)	4.8% (n = 3)
No protein restricted diet but amino acid supplements/GMP	4.8% (n = 2)	0% (n = 0)
No protein restricted diet, no amino acid supplements/GMP	9.5% (n = 4)	0% (n = 0)
BH4 (Sapropterin, Kuvan©) and a protein restricted diet with amino acid supplements/GMP	0% (n = 0)	6.5% (n = 4)
BH4 (Sapropterin, Kuvan©) and a protein restricted diet without amino acid supplements/GMP	0% (n = 0)	1.6% (n = 1)
BH4 (Sapropterin, Kuvan©), no protein restricted diet but amino acid supplements/GMP	0% (n = 0)	1.6% (n = 1)
BH4 (Sapropterin, Kuvan©), no protein restricted diet, no amino acid supplements/GMP	0% (n = 0)	4.8% (n = 3)
Other, namely…	0% (n = 0)	1.6% (n = 1)^e^

^a^Please note n = 56 of the responses were parents, n = 4 of the responses were grandparents who play a significant role in PKU care, n = 1 of the responses was a PKU patient and a parent of a PKU child, for n = 1 response the exact relationship to the PKU patient was unclear

^b^Range 18–62 years, without 1 outlier of 8 years

^c^Range 0–24 years, without 1 outlier of 60 years

^d^n = 49;

^e^Both parent and child use a protein restricted diet with amino acid supplements

Twenty-one Dutch patients were treated in a CE, with 37 patients treated in a general metabolic treatment centre only. The 37 Dutch patients treated in a general metabolic centre were asked if they would like to visit a CE, and what they would expect from a CE. The 21 Dutch patients who were treated in a centre recently appointed as a CE by the Dutch minister of Health, Welfare and Sport, were asked if they now have different expectations of their visits. The UK (n = 33) and German (n = 13) participants were asked to ‘picture’ both situations. So, all UK and German participants ‘pictured’ being treated in a general metabolic centre and were asked if they would visit a CE, and all UK and German participants ‘pictured’ that their treatment centre would be appointed as a CE. As the Dutch patients only answered the questions specific for their circumstance, of a total of 104 participants, 67 (21 Dutch, with all 33 UK and all 13 German) participants answered questions (pictured) about being treated in a CE (Fig. 1). A total of 83 (37 Dutch, with all 33 UK and all 13 German) participants answered questions (pictured) about being treated in a general metabolic treatment centre when also a CE would be available (Fig. 1).

All the answer options are provided as Additional files, with the most relevant data discussed here.

### General expectations

All of the 104 participants considered they would benefit from a CE. Most participants thought that patients as well as health care providers would benefit from a CE (Additional file 2: Table 1). Most of the participants agreed a CE is responsible for maintaining and sharing knowledge (86%, n = 89), developing guidelines (83%, n = 86) and performing scientific research (70%, n = 73, Additional file 3: Table 2. Approximately 65% (n = 68) agreed that new treatments should first start in a CE. Most participants considered a CE should pro-actively collaborate with PKU patient associations and develop patient information (both 65%, n = 68, Additional file 4: Table 3) (Table 2).

**Table 2:** Professions correspondents would like to see when they visit a centre of expertise or when they receive an extensive review

	When you visit a centre of expertise (n = 83) (%)	When you receive an extensive review (n = 67) (%)
Physician	84.3	83.6
Dietician	77.1	79.1
Psychologist (for discussing any problems or mental health issues)	48.2	50.7
Social worker	15.7	10.4
Neuropsychologist (for brain function tests such as IQ)	51.8	59.7
Not applicable, I am not interested in visiting a PKU centre of expertise/an extensive review	3.6	4.5
Other namely	7.2	7.5

Multiple answer options were possible

^1^Other namely: Geneticist, Dexa-scan, obstetrician, sports physician

### Content of visit

When participants ‘pictured’ their treatment in their own hospital as an appointed CE (n = 67), 25% (n = 17) did not expect their hospital visits to change in contrast to 61% (n = 41) who expected more extensive examinations and 64% (n = 43) who expected better facilities (Additional file 7: Table 6). Most participants expected to see a physician (84%, n = 56) and dietician (79%, n = 53) during this extensive examination. Many participants also requested a psychology review (51%, n = 34) and a neuropsychology assessment with neuropsychometric testing (60%, n = 40). A social worker was less frequently mentioned (10%, n = 7, Table 2).

When participants ‘pictured’ visiting a CE, but continuing day to day care in their regular metabolic centre (n = 83) a similar response was seen. Many participants also mentioned they would like to see a physician and dietician when visiting a CE and requested a psychology review and neuropsychology assessment (Table 2). Seventy-eight per cent (n = 65) considered CE physicians and dieticians should have a higher level of knowledge than professionals in their general metabolic treatment centres (Additional file 8: Table 7).

In general, most participants expected to be updated regarding new research developments, new treatments and new dietary products (including protein substitutes) during a CE visit or an extensive review (Additional file 8: Table 7 and Additional file 9: Table 8). Also, most participants expected CEs to collaborate with non-CEs (Additional file 8: Table 7).

### Frequency of visits

**Fig. 2:**
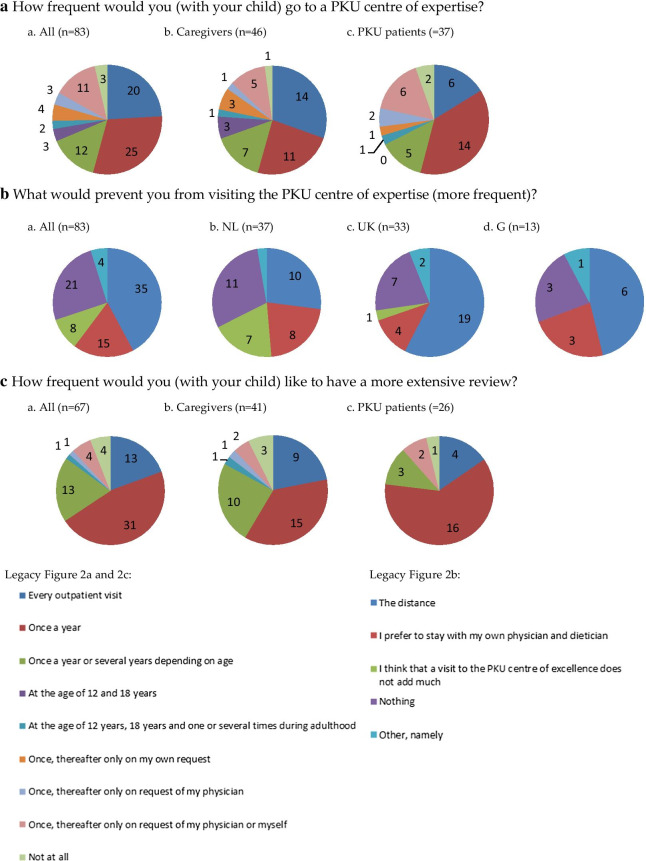
Responders results frequency of visits. G: Germany, NL: the Netherlands, PKU: Phenylketonuria; UK; the United Kingdom

For the participants who ‘pictured’ care in a general metabolic centre (n = 83), the frequency they considered they should visit a CE alongside their own centre varied. Some stated each outpatient visit (24%, n = 20), others annually or bi- annually (45%, n = 37), at a defined patient age (6%, n = 5), one initial visit (22%, n = 18) or not at all (4%, n = 3). Caregivers were more likely to answer every outpatient visit then patients (Fig. 2a). The main barrier preventing more frequent visits was distance to clinic (42%, n = 35). This barrier was reported more frequently by participants from the UK and Germany versus the Netherlands (58%, n = 19; 46%, n = 6; and 27%, n = 10 respectively). Some Dutch participants (19%, n = 7) considered they would not gain from additional CE visits (Fig. 2b).

When participants ‘pictured’ their treatment in their own hospital established as a CE (n = 67), most participants requested a more extensive review annually or bi-annually (66%, n = 44, Fig. 2c).

## Discussion

This is the first survey that evaluated the expectations of patients with PKU and caregivers about CEs. As numerous University centres have treated PKU for many years, we expected that the professionals and/or the patients would not see the advantages of travelling to a CE. Interestingly, only 3 of 83 respondents said they would not visit a CE (Fig. 1a). In addition, when participants ‘pictured’ their treatment in their own hospital as an appointed CE (n = 67), 61% (n = 41) expected more extensive examinations than they receive now.

Before discussing our findings in more detail, some limitations of this study need to be addressed. These small numbers of answers may not represent the aspirations and ideas of the total PKU society. Patients and caregivers who are less interested most likely did not complete the survey. The responder-rate was lower in the relatively larger countries UK and Germany. This probably reflects that this topic is of less interest and less understanding as no PKU centres have been officially appointed yet or the research team was not well known within the country.

In general, PKU is not the only rare disorder a metabolic team is responsible for. Considering there are over 500 rare inherited metabolic disorders described (https://rarediseases.info.gov), it is impossible for a centre to be a leader in scientific research and be informed of all new treatment options and developments in all disorders. Therefore, university metabolic centres should choose disorders which they can provide a higher level of expertise. To continue building expertise, it is valuable that the CEs care for and evaluate a large patient cohort, to learn from and with these patients. Consequently, this new knowledge can be shared with the treatment centres, so that all are able to deliver a high patient care standard and simultaneously use their time to focus on the disorders for which their research and international experience fulfil the criteria of a CE. A study by Camfield et al. confirmed that centrally coordinated specialized care is beneficial for patients as this approach was associated with significant better control of blood Phe, more regular supervisory visits and less frequent discontinuation of diet compared with a decentralized care model [7]. Regarding research output and impact Hannemann-Weber et al. [5] found a positive association with the operational experience, expressed as number of patients treated, and supported the establishment of CEs. Including discussions about clinical studies as part of regular conversations during clinic visits is an important recruitment strategy for rare disease studies [8]. As CEs are leaders in knowledge about the latest developments and research, reviewing more patients with PKU can increase both research participation and output and then further expand knowledge. Together we need to build a structure of CEs within countries, to increase the quality of care.

The frequency patients and caregivers defined they would like to attend a CE along to their own metabolic centre differed. Only 17% of 83 respondents who ‘pictured’ to visit a CE expected to no longer visit their own hospital (Additional file 8: Table 7). In contrast 13 adults of the 37 patients with PKU answered they would like to visit a CE annually (Fig. 2a), which may be the same frequency as they visit their own general metabolic treatment centre. In practice there are barriers when patients visit a CE. In the survey participants identified distance from the CE. A practical solution could be that the CE undertakes ‘outreach’ clinics at different locations, or in the form of video/virtual consultations [9]. Even web-based neuropsychological test batteries are available [10–12]. For discussing new developments and/or research recruitment, patient webinars could be organised.

Also, most CEs will not immediately have the capacity to see extra patients with PKU. The CE commencement of a new (drug) therapy, which requires expertise and additional monitoring could be the start of accepting additional patient referrals. Approximately 65% of all participants (n = 104) agreed that new treatments should first start in CEs. This is likely to result in increased efficiency as training and explanations will be given by a team who have already developed expertise. Furthermore, adult patients with PKU commonly have many outstanding questions regarding treatment, symptoms, and outcome, and would value from care in a CE. Also, patients and caregivers who choose to visit a CE should always be referred as a matter of right.

Another interesting point was that two thirds of the participants who ‘pictured’ their care was already established in a CE expected a more extensive review. In general, about half of all participants requested to see a psychology and neuropsychologist (Table 2). In most centres in the Netherlands, Germany and UK routine neuropsychological testing and psychological guidance is not part of their usual care package, even though it is a recommendation of the European PKU guidelines [13, 14]. This participant response shows there is patient demand for this service as previously reported [2, 15]. This is understandable as PKU is a brain disease and only blood phenylalanine is measured as a surrogate marker of outcome. It is established that only part of the neurocognitive outcome is explained by the phenylalanine concentrations [16, 17]. To help solve which mechanisms influence cerebral disturbances and cognitive reserve in adults and elderly patients with PKU, and identify which biomarkers optimally predict these mechanisms, CEs are crucial [18–22]. But more importantly, participants requesting for neuropsychological testing underlines the patients need to explore brain function. If there is more data available about individual neurocognitive outcome, it will help direct the best treatment strategy.

## Conclusions

In this study most participants expected that assigning CEs will change the structure and content of PKU care to some extent. Most patients would like to visit a CE or expect the content of their consultations to change when they are already treated in a CE. It is important that patient societies discuss this topic with their members as this is at least partially demand and supply driven. For health care centres it is worthwhile to think about the structure to keep building expertise in PKU care in order to strive for an optimal patient outcome.

## Supplementary Information

**Additional file 1:** Questionnaire - How do you see the future in terms of treatment for PKU?

**Additional file 2: Table 1.** Answers of the correspondents to the question: Who do you think can benefit from the PKU centres of expertise in your country that are affiliated with the European collaboration?

**Additional file 3: Table 2.** Answers of the correspondents to the question: What do you expect from the PKU centres of expertise and not so much from the other hospitals?

**Additional file 4: Table 3.** Answers of the correspondents to the question: How would you like to receive information about participation in scientific research?

**Additional file 5: Table 4.** Answers of the correspondents to the question: How would you like to receive information about new developments?

**Additional file 6: Table 5.** Answers of the correspondents to the question: I would participate in scientific research if...

**Additional file 7: Table 6.** Answers of the correspondents to the question: The treatment of you or your child is located in a hospital which is now officially a PKU centre of expertise. What do you expect of your hospital visits in the future?

**Additional file 8: Table 7.** Answers of the correspondents to the question: What do you expect when you or your child visits a PKU centre of expertise?

**Additional file 9: Table 8.** Answers of the correspondents to the question: The treatment of you or your child is located in a hospital which is now officially a PKU centre of expertise. What do you expect when receiving an extensive review?

## Data Availability

All data generated or analysed during this study are included in this published article (and its supplementary information files).

## Abbreviations

CE: Centre of expertise
ERN: European reference networks
Phe: Phenylalanine
PKU: Phenylketonuria
UK: United Kingdom

## References

[1] Blau N, van Spronsen FJ, Levy HL (2010). Phenylketonuria. Lancet.

[2] Hagedorn TS (2013). Requirements for a minimum standard of care for phenylketonuria: the patients' perspective. Orphanet J Rare Dis.

[3] EUCERD, Recommendations on quality criteria for centres of expertise for rare diseases in member states 2011. www.eucerd.eu.

[4] Taruscio D (2014). Centres of Expertise and European Reference Networks: key issues in the field of rare diseases. EUCERD Recomm Blood Transfus.

[5] Hannemann-Weber H, Kessel M, Schultz C (2012). Research performance of centers of expertise for rare diseases—the influence of network integration, internal resource access and operational experience. Health Policy.

[6] Plockinger U, Ziagaki A (2019). The German National Action League for people with rare diseases: translating the three tiers center system into active co-operation, a one center experience. Orphanet J Rare Dis.

[7] Camfield CS (2004). Optimal management of phenylketonuria: a centralized expert team is more successful than a decentralized model of care. J Pediatr.

[8] DeWard SJ (2014). Practical aspects of recruitment and retention in clinical trials of rare genetic diseases: the phenylketonuria (PKU) experience. J Genet Couns.

[9] Donaghy E (2019). Acceptability, benefits, and challenges of video consulting: a qualitative study in primary care. Br J Gen Pract.

[10] Honarmand K (2019). Feasibility of a web-based neurocognitive battery for assessing cognitive function in critical illness survivors. PLoS ONE.

[11] Silverstein SM (2007). Development and validation of a World-Wide-Web-based neurocognitive assessment battery: WebNeuro. Behav Res Methods.

[12] Backx, R., et al. *Bringing Home Cognitive Assessment: Initial Validation of Unsupervised Web-based Cognitive Testing on the Cambridge Neuropsychological Test Automated Battery (CANTAB) using a within-subjects counterbalanced design*. Journal of Medical Internet Research 2020. 10.2196/16792 PMC7435628 32749999

[13] van Spronsen FJ (2017). Key European guidelines for the diagnosis and management of patients with phenylketonuria. Lancet Diabetes Endocrinol.

[14] van Wegberg AMJ (2017). The complete European guidelines on phenylketonuria: diagnosis and treatment. Orphanet J Rare Dis.

[15] Ford S, O'Driscoll M, MacDonald A (2018). Living with Phenylketonuria: Lessons from the PKU community. Mol Genet Metab Rep.

[16] Jahja R (2017). Long-term follow-up of cognition and mental health in adult phenylketonuria: a PKU-COBESO study. Behav Genet.

[17] Jahja R (2014). Neurocognitive evidence for revision of treatment targets and guidelines for phenylketonuria. J Pediatr.

[18] de Groot MJ (2015). Phenylketonuria: brain phenylalanine concentrations relate inversely to cerebral protein synthesis. J Cereb Blood Flow Metab.

[19] Vaclavik J (2018). Structural elucidation of novel biomarkers of known metabolic disorders based on multistage fragmentation mass spectra. J Inherit Metab Dis.

[20] van Vliet D (2015). Large neutral amino acid supplementation exerts its effect through three synergistic mechanisms: proof of principle in phenylketonuria mice. PLoS ONE.

[21] Wasserstein MP (2006). Cerebral glucose metabolism in adults with early treated classic phenylketonuria. Mol Genet Metab.

[22] Yano S, Moseley K, Azen C (2014). Melatonin and dopamine as biomarkers to optimize treatment in phenylketonuria: effects of tryptophan and tyrosine supplementation. J Pediatr.

