# Supplementary material for: Patient’s thoughts and expectations about centres of expertise for PKU
Source: Orphanet J Rare Dis. 2021 Jan 6;16:2. doi: 10.1186/s13023-020-01647-7 (PMC7789756; doi:10.1186/s13023-020-01647-7)
Supplement: Supplementary file 1 — Additional file 1: Questionnaire - How do you see the future in terms of treatment for PKU? [file 13023_2020_1647_MOESM1_ESM.doc]

**Additional File 1. Questionnaire - How do you see the future in terms of treatment for PKU?**

**PART 1: GENERAL INFORMATION**

1. Which of the following statements applies to you?

- I am a PKU patient
- I am a parent / carer of a PKU patient
- I am a grandparent of a PKU patient
- Other, namely… ___________________________________________________

2. What is your gender?

- Male
- Female

3. What is your current age as a PKU patient / the age of your child with PKU

[*age*] years

4. The current treatment of you / your child is the following:

- A protein restricted diet with amino acid supplements or alternative protein substitutes
- A protein restricted diet without amino acid supplements or alternative protein substitutes
- No protein restricted diet but amino acid supplements or alternative protein substitutes
- No protein restricted diet, no amino acid supplements or alternative protein substitutes
- BH4 (Sapropterin, Kuvan©) and a protein restricted diet with amino acid supplements or alternative protein substitutes
- BH4 (Sapropterin, Kuvan©) and a protein restricted diet without amino acid supplements or alternative protein substitutes
- BH4 (Sapropterin, Kuvan©), no protein restricted diet but amino acid supplements or alternative protein substitutes
- BH4 (Sapropterin, Kuvan©), no protein restricted diet, no amino acid supplements or alternative protein substitutes
- Other, namely… ___________________________________________________

5. In which centre are you / is your child treated?

[*name of centres*]

**END OF PART 1.**

**PART 2: EXPECTATIONS OF CENTRES OF EXPERTISE**

**1. Who do you think can benefit from the PKU centres of expertise in** [*name country***]** **that are affiliated with the European collaboration?**

**NOTE: Multiple answers are possible.**

- All PKU patients (general advice)
- The individual patient (personal advice)
- All healthcare providers (physicians, dieticians, etc.) in all hospitals who treat PKU
- Only healthcare practitioners (physicians, dieticians, etc.) in the PKU centres of expertise
- Researchers
- None
- Other, namely … ___________________________________________________

**2. Below are a number of statements about your expectations from the centres of expertise compared to the other hospitals who treat PKU. So what do you expect from the PKU centres of expertise** and not so much from the other hospitals?

|  | Strongly disagree | Disagree | Neither agree nor disagree | Agree | Strongly agree |
| --- | --- | --- | --- | --- | --- |
| A centre of expertise is responsible for maintaining (international) developments and sharing this (new) knowledge with all hospitals who treat PKU. |  |  |  |  |  |
| A centre of expertise is responsible for developing and disseminating (international) guidelines and care standards (so that treatment is the same in each hospital). |  |  |  |  |  |
| A centre of expertise is responsible for scientific research in the [*name country*]. |  |  |  |  |  |
| A centre of expertise is the first centre in [*name country*] where new treatments are started and monitored. |  |  |  |  |  |
| A centre of expertise takes a lead in collaborating with the PKU patient association. |  |  |  |  |  |
| A centre of expertise establishes and produces patient information in collaboration with the patient association. |  |  |  |  |  |

**3. I would participate in scientific research if...
NOTE: multiple answers are possible.**

- Always
- Only if it does not adversely affect the treatment I am following / my child is following now
- Only if the next generation may benefit
- Only if I or my child can directly benefit from it (for example when investigating a new treatment)
- Only if the instruction, support and necessary tests can take place in my own hospital
- Only if the physician / dietician of my own hospital think it is a good idea
- Only if it is led by the physician / dietician of my own hospital
- Only if at least one PKU centre of expertise in [name of country] is in favor
- Only if at least one PKU centre of expertise in [name of country] leads the study
- Only if the PKU patient association has ‘approved’ the study
- Never

**4. How would you like to receive information about participation in scientific research?
Select the answer that applies the most.**

- I am not interested in this information
- From my physician during an outpatient visit
- Through a separate information letter by post
- Through the magazine of the PKU patient association
- Through a National PKU patients meeting
- Through email
- Through social media
- Through a website (which I can routinely check myself)
- Other, namely… ___________________________________________________

**5. How would you like to receive information about new developments?
Select the answer that applies the most.**

- I am not interested in this information
- From my physician during an outpatient visit
- Through a separate information letter by post
- Through the magazine of the PKU patient association
- Through the National PKU patients meeting
- Through email
- Through social media
- Through a website (which I can routinely check myself)
- Other, namely… ___________________________________________________

**FOR QUESTION 6-9 WE ASK YOU TO IMAGINE THE CENTRE YOU OR YOU CHILD IS TREATED IN WILL NOT BE APPOINTED TO BE A CENTRE OF EXPERTISE. YOUR CENTRE WILL CONTINUE THE TREATMENT OF PKU, BUT DOES NOT AIM TO ACQUIRE KNOWLEDGE AND EXPERIENCE TO SHARE ON (AN INTER)NATIONAL BASIS.**

**!!** IMAGINE YOU OR YOUR CHILD IS TREATED IN A CENTRE WHICH WILL NOT BE A PKU CENTRE OF EXPERTISE **!!
6. How frequent would you (with your child) go to a PKU centre of expertise?
Select the answer that applies the most.**

- Every outpatient visit
- Once a year
- Once a year or several years depending on age
- At the age of 12 and 18 years
- At the age of 12 years, 18 years and one or several times during adulthood
- Once, thereafter only on my own request
- Once, thereafter only on request of my physician
- Once, thereafter only on request of my physician or myself
- Not at all

**!!** IMAGINE YOU OR YOUR CHILD IS TREATED IN A CENTRE WHICH WILL NOT BE A PKU CENTRE OF EXPERTISE **!!**

**7. What would prevent you from visiting the PKU centre of expertise (more frequent)?
Select the answer that applies the most.**

- The distance
- I prefer to stay with my own physician and dietician
- I think that a visit to the PKU centre of expertise does not add much
- Nothing
- Other, namely… ___________________________________________________

**!!** IMAGINE YOU OR YOUR CHILD IS TREATED IN A CENTRE WHICH WILL NOT BE A PKU CENTRE OF EXPERTISE **!!
8. During the visit to a PKU centre of expertise I would like to see:
NOTE: multiple answers are possible.**

- Physician
- Dietician
- Psychologist (for discussing any problems or mental health issues)
- Social worker
- Neuropsychologist (for brain function tests such as IQ)
- Not applicable, I am not interested in visiting a PKU centre of expertise
- Other, namely… ___________________________________________________

**!! IMAGINE YOU OR YOUR CHILD IS TREATED IN A CENTRE WHICH WILL NOT BE A PKU CENTRE OF EXPERTISE !!
9. What do you expect when you or your child visits a PKU centre of expertise?**

|  | Disagree | Neither agree nor disagree | Agree | Not applicable |
| --- | --- | --- | --- | --- |
| I expect a more extensive examination than in I receive in my own hospital |  |  |  |  |
| I expect to be treated in the PKU centre of expertise and to no longer visit my own hospital |  |  |  |  |
| I expect the PKU centre of expertise to discuss the results with me and to communicate the results to my own hospital |  |  |  |  |
| I expect the PKU centre of expertise communicates the results (including the recommendations) to my own hospital and that my own physician discusses these with me |  |  |  |  |
| I expect the PKU centre of expertise receives the lab results and other important data of the last year from my own hospital |  |  |  |  |
| I expect to be updated about new developments in scientific research |  |  |  |  |
| I expect to be updated about new developments in new treatment options |  |  |  |  |
| I expect to be updated about new developments in (low protein foods and) amino acid supplements |  |  |  |  |
| I expect the physician and dietician to have a higher level of knowledge than in other hospitals |  |  |  |  |
| I expect more personal recommendations than I receive in my own hospital |  |  |  |  |
| I expect a wider choice of consulting hours (multiple days a week) |  |  |  |  |
| I expect to meet other PKU patients on the same day |  |  |  |  |
| I expect to be informed about education, social activities and networking with other patients (in addition to the information of my own hospital) |  |  |  |  |
| I expect the possibility of digital video consultations via Skype |  |  |  |  |
| I expect a webpage with information about (new developments in) PKU and where I can ask a question to a physician, dietician or other professional |  |  |  |  |
| I am unsure what to expect |  |  |  |  |

**FOR QUESTION 10-13 WE ASK YOU TO IMAGINE THE CENTRE YOU OR YOU CHILD IS TREATED IN WILL BE APPOINTED TO BE A CENTRE OF EXPERTISE. BESIDES TREATING PKU, YOUR CENTRE AIMS TO ACQUIRE KNOWLEDGE AND EXPERIENCE TO SHARE ON (AN INTER)NATIONAL BASIS.**

**!!** IMAGINE YOU OR YOUR CHILD IS TREATED IN A CENTRE WHICH WILL BE A PKU CENTRE OF EXPERTISE **!!**

**10. The treatment of you or your child is located in a hospital which is now officially a PKU centre of expertise. What do you expect of your hospital visits in the future?
Select the answer that applies the most.**

- I expect the content and duration of the hospital visits do not change
- 2. As a centre of expertise I expect the facilities to be better than they are now (e.g. frequent blood analyzes, longer consultations, more direct contact with experts .)
- 3. I expect during every outpatient visit a more extensive examination than I have been used to so far
- 4. I expect during some outpatient visits a more extensive examination than I have been used to so far
- Combination of 2 and 3
- Combination of 2 and 4

**!!** IMAGINE YOU OR YOUR CHILD IS TREATED IN A CENTRE WHICH WILL BE A PKU CENTRE OF EXPERTISE **!!**

**11. How frequent would you (with your child) like to have a more extensive review?
Select the answer that applies the most.**

- Every outpatient visit
- Once a year
- Once a year or several years depending on age
- At the age of 12 and 18 years
- At the age of 12 years, 18 years and one or several times during adulthood
- Once, thereafter only on my own request
- Once, thereafter only on request of my physician
- Once, thereafter only on request of my physician or myself
- Not at all, I do not expect an extensive review

**!!** IMAGINE YOU OR YOUR CHILD IS TREATED IN A CENTRE WHICH WILL BE A PKU CENTRE OF EXPERTISE **!!**

**12. During an extensive review I would like to see:
NOTE: multiple answers are possible.**

- Physician
- Dietician
- Psychologist (for discussing any problems such as difficulties with the diet)
- Social worker
- Neuropsychologist (for brain function tests such as IQ)
- Not applicable, I am not interested in an extensive review
- Other, namely… ___________________________________________________

**!!** IMAGINE YOU OR YOUR CHILD IS TREATED IN A CENTRE WHICH WILL BE A PKU CENTRE OF EXPERTISE **!!**

**13. What do you expect when you or your child has an extensive review at the centre of expertise?**

|  | Disagree | Neither agree nor disagree | Agree | Not applicable |
| --- | --- | --- | --- | --- |
| I expect to be updated about new developments in scientific research |  |  |  |  |
| I expect to be updated about new developments in new treatment options |  |  |  |  |
| I expect to be updated about new developments in (low protein foods and) amino acid supplements or alternative protein substitutes. |  |  |  |  |
| I expect more personal recommendations than I receive during regular outpatient visits |  |  |  |  |
| I expect a wider range of consulting hours (multiple days a week) |  |  |  |  |
| I expect to meet other PKU patients that day |  |  |  |  |
| I expect to be informed about education, social activities and networking with other patients (in addition to the information of the regular outpatient visits) |  |  |  |  |
| I expect the possibility of digital video consultations via Skype |  |  |  |  |
| I expect a webpage with information about (new developments in) PKU and where I can ask a question to a physician, specialist or dietician |  |  |  |  |
| I am unsure what to expect |  |  |  |  |

**14. Do you have any other comments**?

|  |
| --- |

**END OF SURVEY.**
