# Supplementary material for: Patient’s thoughts and expectations about centres of expertise for PKU
Source: Orphanet J Rare Dis. 2021 Jan 6;16:2. doi: 10.1186/s13023-020-01647-7 (PMC7789756; doi:10.1186/s13023-020-01647-7)
Supplement: Supplementary file 2 — Additional file 2: Table 1. Answers of the correspondents to the question: Who do you think can benefit from the PKU centres of expertise in your country that are affiliated with the European collaboration? [file 13023_2020_1647_MOESM2_ESM.docx]

**Additional file 2** 

**Table 1. Answers of the correspondents to the question: Who do you think can benefit from the PKU centres of expertise in your country that are affiliated with the European collaboration?** Multiple answer options were possible

|  | **Total  (n= 104)** | **NL  (n=58)** | **UK  (n=33)** | **GE  (n=13)** |
| --- | --- | --- | --- | --- |
| All PKU patients (general advice) | 91,3% | 91,4% | 90,9% | 92,3% |
| The individual patient (personal advice) | 74,0% | 77,6% | 60,6% | 92,3% |
| All healthcare providers (physicians, dieticians, etc.) in all hospitals who treat PKU | 76,0% | 82,8% | 72,7% | 53,8% |
| Only healthcare practitioners (physicians, dieticians, etc.) in the PKU centres of expertise | 35,6% | 55,2% | 6,1% | 23,1% |
| Researchers | 68,3% | 77,6% | 54,5% | 61,5% |
| No one | 0,0% | 0,0% | 0,0% | 0,0% |
| Other namely | 6,7% | 3,4% | 15,2% | 0,0% |

*Other namely: family members, patient advocacy groups.*
