# Supplementary material for: Patient’s thoughts and expectations about centres of expertise for PKU
Source: Orphanet J Rare Dis. 2021 Jan 6;16:2. doi: 10.1186/s13023-020-01647-7 (PMC7789756; doi:10.1186/s13023-020-01647-7)
Supplement: Supplementary file 3 — Additional file 3: Table 2. Answers of the correspondents to the question: What do you expect from the PKU centres of expertise and not so much from the other hospitals? [file 13023_2020_1647_MOESM3_ESM.docx]

**Additional file 3**

**Table 2. Answers of the correspondents to the question: What do you expect from the PKU centres of expertise and not so much from the other hospitals?** Six statements**.**

|  | **Total (n=104)*** | | | | |
| --- | --- | --- | --- | --- | --- |
|  | SD | D | N | A | SA |
| A centre of expertise is responsible for maintaining (international) developments and sharing this (new) knowledge with all hospitals who treat PKU. | 3,8% | 4,8% | 4,8% | 26,0% | 59,6% |
| A centre of expertise is responsible for developing and disseminating (international) guidelines and care standards (so that treatment is the same in each hospital). | 4,8% | 2,9% | 8,7% | 34,6% | 48,1% |
| A centre of expertise is responsible for scientific research in your country. | 2,9% | 9,6% | 16,3% | 33,7% | 36,5% |
| A centre of expertise is the first centre in your country where new treatments are started and monitored. | 7,7% | 13,5% | 12,5% | 30,8% | 34,6% |
| A centre of expertise takes a lead in collaborating with the PKU patient association. | 6,7% | 10,6% | 16,3% | 37,5% | 27,9% |
| A centre of expertise establishes and produces patient information in collaboration with the patient association. | 3,8% | 6,7% | 23,1% | 37,5% | 27,9% |

*SD = strongly disagree, D = disagree, N = neither agree nor disagree, A = agree SA = strongly agree
*1 participant did not answer this question (1.0%)*
