# Supplementary material for: Patient’s thoughts and expectations about centres of expertise for PKU
Source: Orphanet J Rare Dis. 2021 Jan 6;16:2. doi: 10.1186/s13023-020-01647-7 (PMC7789756; doi:10.1186/s13023-020-01647-7)
Supplement: Supplementary file 4 — Additional file 4: Table 3. Answers of the correspondents to the question: How would you like to receive information about participation in scientific research? [file 13023_2020_1647_MOESM4_ESM.docx]

**Additional file 4

Table 3. Answers of the correspondents to the question: How would you like to receive information about participation in scientific research?**

|  | **Total (n=104)** |
| --- | --- |
| I am not interested in this information | 1,0% |
| From my physician during an outpatient visit | 41,3% |
| Through a separate information letter by post | 31,7% |
| Through the magazine of the PKU patient association | 4,8% |
| Through a National PKU patients meeting | 0,0% |
| Through email | 17,3% |
| Through social media | 0,0% |
| Through a website (which I can routinely check myself) | 1,9% |
| Other, namely | 1,9%*^* |

** remark: in the NL questionnaire there was no option “through email”, “social media” or” website”. However 4 responded mail through the answer other namely. ^One participant commented: a combination of above.*
