# Supplementary material for: Patient’s thoughts and expectations about centres of expertise for PKU
Source: Orphanet J Rare Dis. 2021 Jan 6;16:2. doi: 10.1186/s13023-020-01647-7 (PMC7789756; doi:10.1186/s13023-020-01647-7)
Supplement: Supplementary file 6 — Additional file 6: Table 5. Answers of the correspondents to the question: I would participate in scientific research if... [file 13023_2020_1647_MOESM6_ESM.docx]

**Additional file 6 

Table 5. Answers of the correspondents to the question: I would participate in scientific research if...** Multiple answer options were possible

|  | **Total (n=104)** |
| --- | --- |
| Always | 14,4% |
| Only if it does not adversely affect the treatment I am following / my child is following now | 63,5% |
| Only if the next generation may benefit | 18,3% |
| Only if I or my child can directly benefit from it (for example when investigating a new treatment) | 31,7% |
| Only if the instruction, support and necessary tests can take place in my own hospital | 17,3% |
| Only if the physician / dietician of my own hospital think it is a good idea | 31,7% |
| Only if it is led by the physician / dietician of my own hospital | 4,8% |
| Only if at least one PKU centre of expertise in your country is in favor | 14,4% |
| Only if at least one PKU centre of expertise in your country leads the study | 10,6% |
| Only if the PKU patient association has ‘approved’ the study | 13,5% |
| Never | 1,9% |
