# Supplementary material for: Patient’s thoughts and expectations about centres of expertise for PKU
Source: Orphanet J Rare Dis. 2021 Jan 6;16:2. doi: 10.1186/s13023-020-01647-7 (PMC7789756; doi:10.1186/s13023-020-01647-7)
Supplement: Supplementary file 7 — Additional file 7: Table 6. Answers of the correspondents to the question: The treatment of you or your child is located in a hospital which is now officially a PKU centre of expertise. What do you expect of your hospital visits in the future? [file 13023_2020_1647_MOESM7_ESM.docx]

**Additional file 7

Table 6. Answers of the correspondents to the question: The treatment of you or your child is located in a hospital which is now officially a PKU centre of expertise. What do you expect of your hospital visits in the future?**

|  | **Total (n=67)** |
| --- | --- |
| 1. I expect the content and duration of the hospital visits do not change | 25,4% |
| 2. As a centre of expertise I expect the facilities to be better than they are now (e.g. frequent blood analyzes, longer consultations, more direct contact with experts .) | 13,4% |
| 3. I expect during every outpatient visit a more extensive examination than I have been used to so far | 4,5% |
| 4. I expect during some outpatient visits a more extensive examination than I have been used to so far | 6,0% |
| Combination of 2 and 3 | 22,4% |
| Combination of 2 and 4 | 28,4% |
