# Supplementary material for: Patient’s thoughts and expectations about centres of expertise for PKU
Source: Orphanet J Rare Dis. 2021 Jan 6;16:2. doi: 10.1186/s13023-020-01647-7 (PMC7789756; doi:10.1186/s13023-020-01647-7)
Supplement: Supplementary file 8 — Additional file 8: Table 7. Answers of the correspondents to the question: What do you expect when you or your child visits a PKU centre of expertise? [file 13023_2020_1647_MOESM8_ESM.docx]

**Additional file 8

Table 7. Answers of the correspondents to the question: What do you expect when you or your child visits a PKU centre of expertise?**

|  | **Total (n=83)** | | | | |
| --- | --- | --- | --- | --- | --- |
|  | D | | N | A | NA |
| I expect a more extensive examination than in I receive in my own hospital, | | 4,8% | 15,7% | 75,9% | 3,6% |
| I expect to be treated in the PKU centre of expertise and to no longer visit my own hospital | | 55,4% | 24,1% | 16,9% | 3,6% |
| I expect the PKU centre of expertise to discuss the results with me and to communicate the results to my own hospital | | 6,0% | 4,8% | 84,3% | 4,8% |
| I expect the PKU centre of expertise communicates the results (including the recommendations) to my own hospital and that my own physician discusses these with me | | 12,0% | 21,7% | 62,7% | 3,6% |
| I expect the PKU centre of expertise receives the lab results and other important data of the last year from my own hospital | | 3,6% | 14,5% | 75,9% | 6,0% |
| I expect to be updated about new developments in scientific research | | 2,4% | 4,8% | 88,0% | 4,8% |
| I expect to be updated about new developments in new treatment options | | 1,2% | 3,6% | 90,4% | 4,8% |
| I expect to be updated about new developments in (low protein foods and) amino acid supplements | | 2,4% | 7,2% | 88,0% | 2,4% |
| I expect the physician and dietician to have a higher level of knowledge than in other hospitals | | 6,0% | 9,6% | 78,3% | 6,0% |
| I expect more personal recommendations than I receive in my own hospital | | 14,5% | 34,9% | 47,0% | 3,6% |
| I expect a wider choice of consulting hours (multiple days a week) | | 8,4% | 31,3% | 55,4% | 4,8% |
| I expect to meet other PKU patients on the same day | | 15,7% | 45,8% | 30,1% | 8,4% |
| I expect to be informed about education, social activities and networking with other patients (in addition to the information of my own hospital) | | 12,0% | 36,1% | 45,8% | 6,0% |
| I expect the possibility of digital video consultations via Skype | | 15,7% | 48,2% | 31,3% | 4,8% |
| I expect a webpage with information about (new developments in) PKU and where I can ask a question to a physician, dietician or other professional | | 1,2% | 22,9% | 71,1% | 4,8% |
| I am unsure what to expect* | | 12,0%* | 16,9%* | 7,2%* | 19,3%* |

*D = disagree, N = neither agree nor disagree, A = agree NA = not applicable
* 37 participants (44.6%) did not answer this question, as this question was not included in the Dutch survey.*
