# Supplementary material for: Patient’s thoughts and expectations about centres of expertise for PKU
Source: Orphanet J Rare Dis. 2021 Jan 6;16:2. doi: 10.1186/s13023-020-01647-7 (PMC7789756; doi:10.1186/s13023-020-01647-7)
Supplement: Supplementary file 9 — Additional file 9: Table 8. Answers of the correspondents to the question: The treatment of you or your child is located in a hospital which is now officially a PKU centre of expertise. What do you expect when receiving an extensive review? [file 13023_2020_1647_MOESM9_ESM.docx]

**Additional file 9

Table 8. Answers of the correspondents to the question: The treatment of you or your child is located in a hospital which is now officially a PKU centre of expertise. What do you expect when receiving an extensive review?**

|  | **Total (n=67)** | | | | |
| --- | --- | --- | --- | --- | --- |
|  | D | | N | A | NA |
| I expect to be updated about new developments in scientific research | | 0% | 6,0% | 91,0% | 3,0% |
| I expect to be updated about new developments in new treatment options | | 0% | 1,5% | 92,5% | 6,0% |
| I expect to be updated about new developments in (low protein foods and) amino acid supplements or alternative protein substitutes. | | 0% | 6,0% | 92,5% | 1,5% |
| I expect more personal recommendations than I receive during regular outpatient visits | | 7,5% | 17,9% | 70,1% | 4,5% |
| I expect a wider range of consulting hours (multiple days a week) | | 13,4% | 26,9% | 56,7% | 3,0% |
| I expect to meet other PKU patients that day | | 20,9% | 37,3% | 37,3% | 4,5% |
| I expect to be informed about education, social activities and networking with other patients (in addition to the information of the regular outpatient visits) | | 7,5% | 26,9% | 59,7% | 6,0% |
| I expect the possibility of digital video consultations via Skype | | 23,9% | 35,8% | 32,8% | 7,5% |
| I expect a webpage with information about (new developments in) PKU and where I can ask a question to a physician, specialist or dietician | | 9,0% | 11,9% | 76,1% | 3,0% |
| I am unsure what to expect* | | 14,9% | 19,4% | 9,0% | 23,9% |

*D = disagree, N = neither agree nor disagree, A = agree NA = not applicable
* 22 participants (32,8%) , of which 21 Dutch participants, did not answer this question, as this question was not included in the Dutch survey.*
